# Editorial

**DOI:** 10.1007/s00726-022-03164-2

**Published:** 2022-04-29

**Authors:** Dimitrios Tsikas

**Affiliations:** Hannover Medical School, Hannover, Germany

## Post-translational modifications (PTM): analytical approaches, signaling, physiology and pathophysiology—Part II

**Fig. 1:**
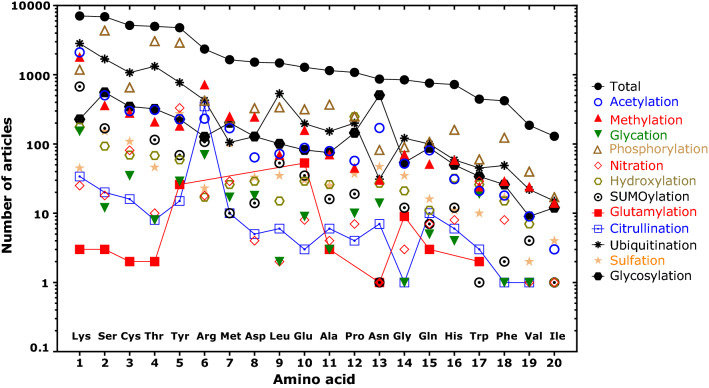
Number of articles reported in the PubMed databank (https://pubmed.ncbi.nlm.nih.gov) for canonical amino acids and some of their post-translational modifications (assessed on 17 March 2022). The total number was obtained by using the term “post-translational modifications” in combination with the name of each amino acid, for instance “lysine”. The number of articles for each PTM was obtained by using the term “post-translational modifications” in combination with the name of each amino acid, for instance “lysine”, and with the name of the PTM, for instance “acetylation”. Relative “maxima” highlight the specificity of some PTMs, such as methylation of Lys and Arg, citrullination of Arg, and glutamylation of Glu. Note the decadic logarithmic scale on the *y*-axis. The order of amino acids on the *x*-axis originates from the number of total articles

Post-translational modifications (PTMs) are since decades in the focus of interest of scientists from various disciplines. Search in the PubMed databank (https://pubmed.ncbi.nlm.nih.gov) using the term “post-translational modifications” resulted in 87,277 papers in the period from 1946 until to date (March 2022) with a current publication rate of more than 5000 articles per year over the last decade. PTMs occur virtually on all kinds of amino acid residues in numerous proteins and are mostly catalyzed by means of specific enzymes. Among the prominent amino acids that undergo PTMs are lysine, arginine, proline, cysteine, threonine, serine, tyrosine, and glutamate. Their PTMs include acetylation (43,787 articles), methylation (4593 articles), and glycation (4684 articles) in addition to the most widely occurring PTMs phosphorylation (15,096 articles) and ubiquitination (9882 articles) (Fig. 1). PTMs change the physicochemical properties of proteins and may have far-reaching consequences for health and disease. Citrullination is specific to arginine residues (505 articles) and is considered to give rise to new antigenic epitopes leading to the generation of autoantibodies and to play a particular role in rheumatoid diseases (Catrina et al. 2021). Hypusination is a unique two-step enzymatic PTM that occurs exclusively on a lysine residue of the eukaryotic initiation factor eIF5a (Park et al. 1981). Advanced glycation end-products (AGEs) belong to the non-enzymatically generated PTMs and serve as useful markers of carbohydrate metabolism notably in diabetes (Nagai et al. 2014).

Amino Acids, a journal dedicated to amino acid, peptide and protein research, organized a special issue on PTMs. The current issue of Amino Acids is entirely dedicated to the second part of the special issue. It includes seven review articles, six original research papers, and a protocol article, written by leading scientists in the area of PTMs.

Dr. M.H. Park’s group was the first to report on the identification of hypusine in a protein from human lymphocytes and of spermidine as its biosynthetic precursor (Park et al. 1981). In this PTM, the putrescine part of the polyamine spermidine is transferred to the terminal amine group of lysine, which is then hydroxylated. Proteolysis of hypusinated eIF5a releases hypusine, a specific marker of this PTM. The group by Dr. Park reviewed in their paper the post-translational formation of hypusine in eIF5A and its implications in human neurodevelopment (Park et al. 2022). Drs. A. Kaiser and E. Agostinelli devoted their review to the hypusinated EIF5A as a feasible drug target for Advanced Medicinal Therapies in the treatment of pathogenic parasites and therapy-resistant tumors (Kaiser and Agostinelli 2022).

*trans*-4-Hydroxy-l-proline is highly abundant in collagen formed from the post-translational hydroxylation of proteins. Proline hydroxylation contributes to the modulation of cell metabolism, growth, development, responses to nutritional and physiological changes, and survival. Dr. G. Wu’s group thoroughly reviewed this topic from several perspectives and summarized the new knowledge of hydroxyproline’s biochemistry and nutrition aids in improving growth, health and well-being of humans and other animals (Hu et al. 2022).

Dr. Ruse’s group reviewed the biology and analysis of polyglutamylation (Ruse et al. 2022), a specific PTM by which up to 50 glutamate molecules are enzymatically transferred to glutamate residues of certain proteins.

Carbonic anhydrases (CAs) are a family of metallo-enzymes that catalyze a very simple chemical reaction, i.e., the reaction of carbon dioxide (CO_2_) with water. The CA-catalyzed hydratation of CO_2_ belongs to the fasted known reactions in nature and plays several roles in health and disease. This enzyme family is also of particular pharmacological importance. Specific and potent inhibitors of CA activity are highly desired, notably for the treatment of cancer (Supuran 2020; Testa et al. 2022). Di Fiori and colleagues reviewed in their paper the PTMs of CAs that are associated with tumors (Di Fiore et al. 2022).

Carbonylation of proteins is generally considered to be associated with oxidative stress. Estévez and colleagues gave in their review a concise update of protein carbonylation in food and nutrition (Estévez et al. 2022).

de Brevern and Rebehmed reviewed the current status of databases that provide three-dimensional (3D) structural data on PTM sites in proteins (de Brevern and Rebehmed 2022). Databases are important, because they help understand the influence and dynamics of PTMs and are crucial for unraveling underlying processes. Investigations by the authors revealed that currently available databases suffer from multiple problems and the authors conclude that care must be taken when analyzing the PTMs data proteins (de Brevern and Rebehmed 2022).

In the research articles included in the second part of the special issue on PTMs, the development, validation, and use of analytical methods including mass spectrometry in in vitro and in vivo studies on various PTMs including glycation are reported. Marsden and colleagues have identified arginine-methylated proteins in human hair, which could serve as novel cardiovascular biomarkers that can be measured non-invasively (Marsden et al. 2022). Sandberg and colleagues characterized a novel + 70-Da modification in rhGM-CSF expressed in *E. coli* using chemical assays in combination with mass spectrometry (Sandberg et al. 2022). Baskal and colleagues reported the development, validation, and application of GC–MS methods for the simultaneous measurement of several AGEs and amino acids in biological fluids including human and rat urine (Baskal et al. 2022a, b). In a bi-ethnic South African population, similar excretion rates of AGEs and hypusine were measured (Baskal et al. 2022a, c) that suggest no ethnic-dependent PTMs in black and white boys. Katsuka and colleagues investigated aging-associated changes in AGEs including *S*-(2-succinyl)cysteine in mouse tissues (Katsuta et al. 2022). Padilla and colleagues report an in vitro assay of the effect of the lysine oxidation end-product, α-aminoadipic acid, on the redox status and gene expression in probiotic *Lactobacillus reuteri* PL503 (Padilla et al. 2022).

The closely cooperating groups by Drs. I. Dalle-Done (Milan, Italy) and R. Rossi (Siena, Italy) are leading in the area of oxidative stress. The authors provide a detailed protocol that is used in their groups to measure *S*-glutathionylated proteins in humans and animals (Giustarini et al. 2022). *S*-Glutathionylation is a specific PTM of cysteine moieties of cellular and extra-cellular proteins. This protocol should be useful to researchers being interested in thiol-associated oxidative stress.

The PTM special issue (part I and part II) collects only a small fraction of articles. Nevertheless, we hope that this work will contribute to the research of the exciting and challenging topic of PMTs and spur on young scientists.

Prior to closing this special issue, we would like to thank the authors for their contributions and the reviewers for their honorary engagement to improve the papers by providing constructive criticism and helpful suggestions. We express our sincere thanks to all participants for their patience during the COVID-19 pandemic.

## Order of appearance in the special issue PTM part II

### 0) Editorial part II

Tsikas D (2022).

### Reviews

- (1) Park MH, Kar RK, Banka S, Ziegler A, Chung WK (2021) Post-translational formation of hypusine in eIF5A: implications in human neurodevelopment. Amino Acids. https://doi.org/10.1007/s00726-021-03023-6. Online ahead of print. PMID: 34273022.
- (2) Kaiser A, Agostinelli E (2022) Hypusinated EIF5A as a feasible drug target for Advanced Medicinal Therapies in the treatment of pathogenic parasites and therapy-resistant tumors. Amino Acids. https://doi.org/10.1007/s00726-021-03120-6. Online ahead of print. PMID: 35000000 Review.
- (3) Hu S, He W, Wu G (2022) Hydroxyproline in animal metabolism, nutrition, and cell signaling. Amino Acids. https://doi.org/10.1007/s00726-021-03056-x.
- 4) Ruse CI, Hang Chin HG, Pradhan S (2022) Polyglutamylation: biology and analysis. Amino Acids. 10.1007/s00726-022-03146-4
- (5) Di Fiore A, Supuran CT, Scaloni A, De Simone G (2022) Post-translational modifications in tumor-associated carbonic anhydrases. Amino Acids. https://doi.org/10.1007/s00726-021-03063-y. Online ahead of print. PMID: 34436666 Review.
- (6) Estévez M, Díaz-Velasco S, Martínez R (2022) Protein carbonylation in food and nutrition: a concise update. Amino Acids. https://doi.org/10.1007/s00726-021-03085-6. Online ahead of print. PMID: 34669011.
- (7) de Brevern AG, Rebehmed J (2022) Current status of PTMs structural databases: applications, limitations and prospects. Amino Acids. https://doi.org/10.1007/s00726-021-03119-z. Online ahead of print. PMID: 35020020.

### Originals

- (8) Marsden AJ, Riley DRJ, Birkett S, Rodriguez-Barucg Q, Guinn BA, Carroll S, Ingle L, Sathyapalan T, Beltran-Alvarez P (2022) Love is in the hair: arginine methylation of human hair proteins as novel cardiovascular biomarkers. Amino Acids. https://doi.org/10.1007/s00726-021-03024-5. Online ahead of print. PMID: 34181092.
- (9) Sandberg MW, Bunkenborg J, Thyssen S, Villadsen M, Kofoed T (2022) Characterization of a novel + 70-Da modification in rhGM-CSF expressed in E. coli using chemical assays in combination with mass spectrometry. Amino Acids. https://doi.org/10.1007/s00726-021-03004-9. Online ahead of print. PMID: 34453584.
- (10) Baskal S, Bollenbach A, Mels C, Kruger R, Tsikas D (2022a) Development, validation of a GC–MS method for the simultaneous measurement of amino acids, their PTM metabolites and AGEs in human urine, and application to the bi-ethnic ASOS study with special emphasis to lysine. Amino Acids. https://doi.org/10.1007/s00726-021-03031-6. Online ahead of print. PMID: 34251524.
- (11) Baskal S, Büttner P, Werner S, Besler C, Lurz P, Thiele H, Tsikas D (2022b) Profile of urinary amino acids and their post-translational modifications (PTM) including advanced glycation end-products (AGEs) of lysine, arginine and cysteine in lean and obese ZSF1 rats. Amino Acids. https://doi.org/10.1007/s00726-021-03042-3. Online ahead of print. PMID: 34250558.
- (12) Katsuta N, Takahashi H, Nagai M, Sugawa H, Nagai R (2022) Changes in S-(2-succinyl)cysteine and advanced glycation end-products levels in mouse tissues associated with aging. Amino Acids. https://doi.org/10.1007/s00726-022-03130-y. Online ahead of print. PMID: 35166937.
- (13) Padilla P, Andrade MJ, Peña FJ, Rodríguez A, Estévez M (2022) An in vitro assay of the effect of lysine oxidation end-product, α-aminoadipic acid, on the redox status and gene expression in probiotic Lactobacillus reuteri PL503. Amino Acids. https://doi.org/10.1007/s00726-021-03087-4. Online ahead of print. PMID: 34657206.

### Protocols

- (14) Giustarini D, Milzani A, Dalle-Donne I, Rossi R (2022) Measurement of *S*-glutathionylated proteins by HPLC. Amino Acids. https://doi.org/10.1007/s00726-021-03015-6. Online ahead of print. PMID: 34129091.
